# Reflectance Confocal Microscopy of Aging Skin and Skin Cancer

**DOI:** 10.5826/dpc.1103a68

**Published:** 2021-05-20

**Authors:** Stefania Guida, Giovanni Pellacani, Silvana Ciardo, Caterina Longo

**Affiliations:** 1 Dermatology Unit, Department of Surgical, Medical, Dental and Morphological Sciences Related to Transplant, Oncology and Regenerative Medicine, University of Modena and Reggio Emilia, Modena, Italy; 2 Centro Oncologico ad Alta Tecnologia Diagnostica, Azienda Unità Sanitaria Locale-Istituto di Ricovero e Cura a Carattere Scientifico di Reggio Emilia, Reggio Emilia, Italy

**Keywords:** Skin aging, skin cancer, collagen, blood vessels, reflectance confocal microscopy

## Abstract

Skin aging is a complex process that causes morphologic variations. Some of these variations have been hypothesized to be involved in skin cancer development. This paper reviews current knowledge of the features of aged skin as seen with reflectance confocal microscopy (RCM). Basic principles of the technique are described, and the RCM features of healthy skin and skin cancer are briefly discussed. Moreover, the RCM features at different layers of young and elderly skin are described, as are the variations that occur with passing years and in relation to sun exposure that contribute to photoaging and the development of skin cancer. RCM enables the noninvasive evaluation, at quasi-histologic resolution, of aging-related skin changes, some of which are shared with skin cancer; this ability helps avoid skin biopsy. Further research is needed to understand the relation between skin aging and skin cancer development.

## Introduction

Skin aging is a complex biological process leading to skin senescence. Attention to skin aging reflects an increasing consumer demand for products and treatments that can prevent or reverse skin aging signs [1,2]. Importantly, aging is strongly associated with skin cancer development.

Skin aging is influenced by both intrinsic factors, such as chronological age and genetic background, and extrinsic factors, mainly sun exposure, that contribute to “photoaging” [3]. Interestingly, different types of skin photoaging have been identified: atrophic and hypertrophic. Atrophic photoaging is typified by actinic keratosis, seborrheic keratosis, telangiectasia, and a prior diagnosis of skin cancer, while hypertrophic photoaging is characterized by high scores on photoaging severity scales, coarse wrinkles, thickness, and sallowness [4].

UV irradiation, both natural and artificial, is considered the main etiologic factor in photoaging and skin cancer. UV irradiation induces DNA damage (through the formation of cyclobutane pyrimidine dimers), gene mutation, oxidative stress, immunosuppression, and inflammatory responses [5,6]. In fact, painful sunburns have been associated with the development of both melanoma and non-melanoma skin cancers (NMSCs), namely squamous cell carcinoma (SCC) and basal cell carcinoma (BCC) [5,7], while chronic sun exposure is considered the most important causative factor for actinic keratosis and lentigo maligna/lentigo maligna melanoma (LM/LMM) [5,8].

In the past few years, reflectance confocal microscopy (RCM) has been employed as a noninvasive technique to visualize the skin at quasi-histological resolution; it has also been used as an add-on tool in the diagnosis of skin cancer, as a second level of examination after dermoscopy, to improve diagnostic accuracy [9–12]. Additionally, due to the fact that RCM avoids skin biopsy, it has also been applied to the investigation of skin aging signs [13].

This article reviews current knowledge about the use of RCM to study skin aging. In particular, basic principles of the technique are reviewed and the RCM features of different skin cancers are briefly described.

## Basic principles of RCM technology

RCM enables the analysis of healthy skin compartments by providing an in vivo optical biopsy in a totally noninvasive manner. Furthermore, RCM can be repeated in the same area at different times, enabling the assessment of skin variations, such as during treatment monitoring.

A reflectance confocal microscope comprises a point source of light, objective lenses, a condenser, and a point detector. The pinhole collects light from the “in focus” plane. Bright contrast in RCM is related to backscattering. Confocal images are in grayscale, with bright (white) structures having a higher refractive index than their surroundings [14]. Melanin, keratin and collagen are examples of structures that appear bright (white).

Technically, RCM has an axial resolution of 3–5 ***μm*** and a lateral resolution of 1 ***μm***, and it reaches a depth of 250 ***μm***, corresponding to the upper dermis. Two reflectance confocal microscopes are commercially available: a wide-probe microscope (VivaScope 1500, Mavig, Germany) and a handheld model (VivaScope 3000, Mavig, Germany).

The wide-probe RCM instrument has a probe that explores the skin through a disposable plastic window adherent to a metal ring attached to the skin. The result is a sequence of full-resolution, 0.5×0.5 ***μm*** images at a defined depth, acquired and combined to create a mosaic ranging in size from 2×2 mm to 8×8 mm. When inflammatory or physiologic skin conditions are explored, a 3×3 mm VivaCube, composed of 4 mosaics with a 25-μm step, is collected and analyzed. In addition to horizontal mosaics, a vertical VivaStack can be acquired, creating an optical biopsy consisting of a series of 0.5×0.5 high-resolution images at different depths. The number of stacks between the first appearance of a honeycomb pattern and the first appearance of collagen, with a given depth, is used to assess epidermal thickness [13].

The handheld RCM model is a smaller, flexible device that permits the exploration of difficult-to-access areas, such as ears and skin folds. However, the handheld tool does not allow visualization of a large field of view. Only single 0.5×0.5 mm images and VivaStacks can be acquired. The view of larger fields is impaired [1,13].

## Skin morphology over the years

Skin aging involves variations at both the epidermal and dermal levels. These changes can be noninvasively detected with RCM. To detect skin variations that occur with passing time, it is important to understand the morphology of healthy skin as it progressively changes from young skin to elderly skin [1,13,15–23].

### Healthy young skin

RCM can be used to evaluate the epidermis, the dermo- epidermal junction (DEJ) and the upper dermis (Table 1). When analyzing the epidermis, the microscopist first sees the stratum corneum at the top surface. This skin layer is characterized by the presence of large, bright, anucleated cells (corneocytes) that are polygonal, 10–30 mm in size, and have the tendency to form “islands” surrounded by dark areas, corresponding to skin furrows [24].

**Figure 1:**
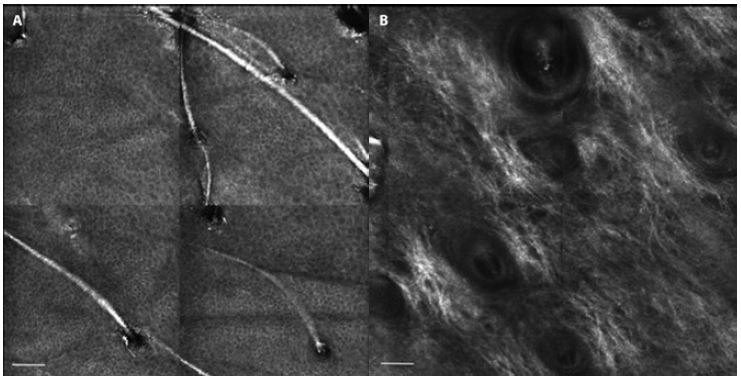
Reflectance confocal micrographs showing morphologic aspects of young skin. (A) Regular honeycomb pattern. (B) Reticular collagen. Scale bars = 100 μm.

Going deeper to the granulosum-spinosum layer, keratinocytes are characterized by dark nuclei and bright cytoplasm. In particular, a grainy appearance is typically observed in cells of the granulosum layer, due to the presence of organelles. The organization of keratinocytes is similar to a honeycomb. Therefore, the typically observed pattern at the epidermal level in people with light skin is called honeycomb pattern (Figure 1A) [24]. In light-skinned people, the keratinocyte contour is brighter than the cytoplasm. Conversely, people with dark skin show a different pattern, called cobblestone pattern, that is the negative of a honeycomb pattern, with pigmented (bright) keratinocytes separated by a dark contour [16].

Going to the basal layer of the epidermis, basal cells appear as cells of the same size (7–12 mm) and shape although they are smaller than the keratinocytes of the upper layers and have high refractivity due to their melanin content [14]. The epidermal thickness can be estimated from the number of stacks from the visualization of the honeycomb pattern to the observation of collagen [13]. Interestingly, melanocytes are not recognized in healthy skin because they share features with keratinocytes in terms of size and amount of melanin [14].

At the DEJ level, basal cells tend to form oval or round bright rings centered by dark dermal papillae, defining the so-called ring pattern [14]. Intuitively, melanin content and skin phototype influence the brightness of keratinocytes: the darker the skin phototype, the brighter the basal cells and rings appear. However, on the face, rings cannot be visualized [16].

In young subjects, the dermis is mainly composed of thin, hyper-reflective reticulated fibers, with a weblike organization (Figure 1B) [16].

Other structures that are visualized are the sebaceous glands, corresponding to hair shafts within hair follicles, and sweat ducts located in the dermis. The face is characterized by several hair follicles creating dark round areas centered by a hair shaft [16].

### Skin aging signs and their correlation with skin cancer

Worldwide, the incidence of skin cancer is progressively increasing, and two main hypotheses have been formulated to explain this fact: increasing sun exposure and population aging [25,26]. Therefore, both chronological aging and photoaging contribute to skin cancer development. It has been shown that they also contribute to skin cancer progression.

#### Epidermal changes

**Figure 2:**
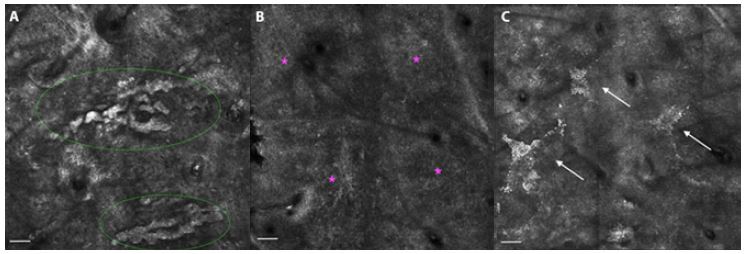
Reflectance confocal microscopy appearance of aged skin. (A) Polycyclic papillary contours (green circles). (B) Atypical honeycomb pattern (pink stars). (C) Mottled pigmentation (white arrows). Scale bars = 100 μm.

With aging, the skin shows progressive changes that can involve all its layers. At the epidermal level, both quantitative and qualitative variations may occur. Accordingly, the skin shows a progressive reduction in epidermal thickness. However, some middle-aged subjects may develop hyperplasia with evidence of polycyclic papillary contours (Figure 2A), histopathologically corresponding to solar lentigo. Therefore, it has been hypothesized that the skin might develop a hyperplastic response to solar damage and then show severe atrophy with progressive thinning [16]. Additionally, there is a change of the overall epidermal surface, showing a prevalent linear furrow pattern with the progressive enlargement of a rhomboidal pattern and then a linear trend without intersecting lines [18,19].

The regular honeycomb pattern, typical of young age, is substituted by an irregular honeycomb pattern during the aging process [13]. The irregular honeycomb pattern shows polygonal keratinocytes with ill-defined cell borders (Figure 2B) and variable size and shape due to varying degrees of keratinocyte atypia, with focal disarray in lesions such as actinic keratosis [9]. Furthermore, melanocytes can be identified in benign melanocytic lesions (eg, melasma, nevus) and malignant melanocytic lesions (eg, melanoma) [16]. In addition, the presence of dyspigmentation at the epidermal level corresponds to the visualization with RCM of bright keratinocytes assembled in the context of a honeycomb pattern, called mottled pigmentation (Figure 2C) [13]. Mottled pigmentation, together with atypical honeycomb pattern, has been shown to be significantly more represented in older adults (aged older than 54 years) [27].

### Dej and dermal changes

The exploration with RCM at the DEJ and superficial dermis level enables the visualization of dermal papillae, blood vessels within each dermal papilla, and collagen (Table 1). In a study of 52 Japanese subjects, reductions in the number and size of dermal papillae were observed in subjects older than 50 years of age compared to a group of 18- to 20-year-old healthy individuals [28].

**Figure 3:**
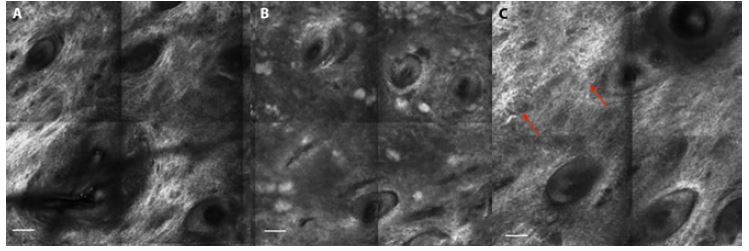
Reflectance confocal micrographs at dermal level. (A) Reticular collagen, typical of young subjects. (B) Coarse collagen. (B) Huddle collagen. (C) Curled fibers in a subject with solar elastosis (red arrows). Scale bars = 100 μm.

Collagen fibers have specific features according to age and photodamage. In aged skin, morphologic variations of collagen include coarse collagen, huddles of collagen, and curled fibers. Coarse collagen is described as a coarse network of large hyporeflecting collagen, huddle collagen appears as large blotches of amorphous material with hyporeflectivity, and curled fibers are visualized as undulated bright structures corresponding to solar elastosis (Figure 3) [13]. Different collagen types have been observed throughout aging in a group of 63 Italian women, with reticular collagen being less evident in subjects older than 35 years and coarse and huddled collagen and curled fibers becoming increasingly represented [13]. Similar results were observed in a group of 44 Brazilian women [29].

#### Skin aging quantification in photo-exposed and non-photo- exposed skin

Different scores have been developed to quantify skin aging. An epidermal disarray score, which ranges from 0 to 9, is calculated from an irregular honeycomb pattern, the epidermal thickness, and a furrow pattern. An epidermal hyperplasia score, which also ranges from 0 to 9, includes the evaluation of mottled pigmentation, polycyclic papillary contours, and epidermal thickness. A collagen alteration score, which ranges from 0 to 12, is estimated from the extent of each collagen type (scored from 0 to 4) multiplied by its coefficient; the coefficient is 3 for curled fibers, 2 for huddles of collagen, 1 for coarse collagen structures, and 0 for thin reticulated collagen [17].

A study evaluated the distribution of these scores, employing RCM images of the face in correspondence of the left malar eminence, in a population of 50 women between 24 and 88 years old [17]. According to the study, the epidermal disarray score was stable until age 65 years and then increased, while the epidermal hyperplasia and collagen scores showed proportional increases with age.

Skin aging scores have also been explored in a larger cohort of 209 French subjects between 74 and 81 years of age [19]. The study analyzed differences between non-photo- exposed (volar arm), chronically photo-exposed (face) and intermittently photo-exposed (dorsal forearm) areas. Photo- exposed areas had significant higher epidermal disarray and epidermal hyperplasia scores than non-photo-exposed areas, while the collagen score was higher in the intermittently photo-exposed skin than the non-photo-exposed skin [19].

Other studies have compared RCM features between non-photo-exposed and photo-exposed skin. These studies identified epidermal, DEJ and dermal changes that were related to chronological aging and that were more evident in photo-exposed areas. These changes included the appearance of linear furrows, mottled pigmentation, atypical honeycomb pattern, irregular ringed pattern, and huddle collagen [19–21].

#### From skin aging to skin cancer

Skin variations occurring with passing years have been related to skin cancer development and progression [4,30,31]. Interestingly, RCM enables the visualization of features shared by aged skin and skin cancer at both the epidermal level, such as atypical cells or atypical honeycomb pattern, and the dermal level, such as dilated vessels or collagen variations.

**Figure 4:**
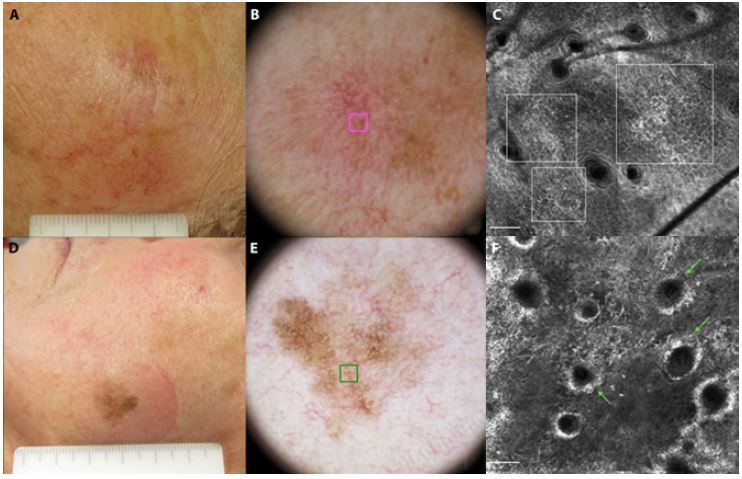
Clinical photograph (A) and dermoscopic image (B) of a pigmented actinic keratosis of the left cheek in a 70-year-old woman. (C) Reflectance confocal micrograph of the area marked by a pink square in B, showing an atypical honeycomb pattern (white squares). (D) Clinical photograph and (E) dermoscopic image of a lentigo maligna on the left cheek of a 64-year-old woman. (F) Reflectance confocal micrograph of the area marked by a green square in E, showing atypical cells (both rounded and dendritic) infiltrating the hair follicle (green arrows). Scale bars = 100 μm.

At the epidermal level, a focal, atypical honeycomb pattern related to chronic sun exposure is typically observed in actinic keratosis (Figure 4, A and B), while a disarranged epidermal pattern and ulceration are commonly observed in SCC [9,32]. Additionally, since melanocytes are not visible in healthy skin, the identification of atypical cells or nests (referred to melanocytes) with RCM contributes to the differential diagnosis of LM/LMM (Figure 4, C and D) with other pigmented macules of the face [33].

At the dermal level, dilated vessels may be observed in NMSC, where they are typically horizontal and branching, surrounding the tumor island in BCC and more irregular in SCC [34,35]. Some other morphologic variations at the dermal level include bundles of lace-like collagen in actinic keratosis and SCC [36] and collagen bundles surrounding dark silhouette in hypopigmented BCC [37,38]. Interestingly, fewer curled fibers, corresponding to solar elastosis, were observed in subjects with atrophic photoaging (more prone to skin cancer) than in patients with hypertrophic photoaging [4,13,23]. Therefore, the lack of solar elastosis in subjects with atrophic photoaging seems to contribute to increased collagen fragmentation, which modifies the dermal microenvironment [4]. Additionally, a lack of solar elastosis has been described in the adjacent skin of melanomas harboring *BRAF* mutations, which are a common finding in melanoma [39].

The correlation between specific epidermal and dermal characteristics and skin photoaging confirms that UV irradiation contributes to cellular atypia; moreover, it seems to support the notion that the dermal microenvironment is an active participant in the formation of skin cancer [4]. Accordingly, increased vascularization has been implicated in skin cancer development because it provides an enriched microenvironment for tumor growth [4,30,31]. The lack of solar elastosis, observed in subjects with atrophic photoaging and in the surrounding skin of melanomas, has been hypothesized as a risk factor for the growth and expansion of skin tumors [4]. Other mechanochemical interactions between aged skin and tumor, which may affect metastasis, have also been described [40].

Interestingly, a different collagen morphology and increased vascularization have been observed in subjects with atrophic photoaging, who are more likely to have skin cancer and to carry melanocortin-1 receptor gene (*MC1R*) polymorphisms [4,22,23]. *MC1R* polymorphisms have been associated with the red hair phenotype, characterized by red hair, freckles, light skin and poor tanning. However, a different *MC1R* genotype might express different phenotypes, sharing the poor tanning ability and tendency to have freckles and light skin [41]. Despite this phenotype, carrying specific *MC1R* polymorphisms increases the risk of melanoma and NMSC [42,43]. This finding seems to provide new insights into the relation between skin photoaging type and the susceptibility to skin cancer, but further studies are needed to explain the complex phenomena leading from skin aging to tumor development and progression.

## Conclusions

RCM facilitates the identification of in vivo features of healthy skin and the recognition of skin aging variations at different skin layers, with histopathologic resolution. Skin aging changes have been shown to correlate with skin cancer development and progression. Interestingly, RCM enables the visualization of features shared by aged skin and skin cancer, at both the epidermal level (eg, atypical cells or atypical honeycomb pattern) and the dermal level (eg, dilated vessels or collagen variations). However, the mechanism that links morphological variations in aging skin to skin cancer needs further investigation.

## Figures and Tables

**Table 1:** Features of Skin Seen with Reflectance Confocal Microscopy

Feature	Description
**Epidermis**	
Regular honeycomb pattern	Polygonal keratinocytes of uniform size and shape, with well-defined borders
Irregular honeycomb pattern	Keratinocytes of variable size and shape and ill-defined borders
Mottled pigmentation	Cluster of bright keratinocytes within a honeycomb pattern
Epidermal thickness	Estimated from the number of stacks (starting from the first visualization of the honeycomb pattern to the presence of collagen)
Furrow aspects	Dark folds between groups of keratinocytes, usually described as rhomboidal in healthy skin, tending to linearity in aged skin
**Dermo-epidermal junction**	
Polycyclic papillary contours	Bulbous projections that can show a variable convoluted arrangement
Sebaceous glands	Annular structures with round or oval shape, centered by hair follicles
**Dermis**	
Thin reticular collagen	Bright, thin, fibrillar structures creating a weblike appearance
Coarse collagen	Coarse filamentous, thick structures with a tendency to be packed
Huddle collagen	Large blotches of amorphous material with hyporefractivity
Curled fibers	Short, thick, undulated fibers with high refractivity
